# Virus production in shallow groundwater at the bank of the Danube River

**DOI:** 10.1371/journal.pone.0306346

**Published:** 2024-08-29

**Authors:** Daniel Pleyer, Christian Griebler, Christian Winter

**Affiliations:** Department of Functional and Evolutionary Ecology, University of Vienna, Vienna, Austria; The University of Akron, UNITED STATES

## Abstract

Viruses play a crucial role in regulating prokaryotic populations, yet their impact on subsurface environments, specifically groundwater habitats, remains poorly understood. In this study, we employed the virus-dilution approach to measure lytic virus production rates in shallow groundwater located near the city of Vienna (Austria) during the period from July−November 2020. Physico-chemical parameters (pH, electrical conductivity, water temperature, concentration of dissolved oxygen), prokaryotic, and viral abundance, and viral decay rates were monitored as well. Our findings revealed low virus-to-prokaryote ratios varying between 0.9−3.9 throughout the study period and a lack of correlation between prokaryotic and viral abundance in groundwater. Virus production rates varied between 9−12% of viral abundance h^**-1**^ in July−August and between 34−36% of viral abundance h^**-1**^ in October−November. Seasonal variations in virus production rates were found to be correlated with electrical conductivity, revealing ~3.5 times higher virus production rates during periods with high electrical conductivity and low groundwater recharge in October−November compared to July−August with higher groundwater recharge and lower electrical conductivity. Our data indicate that groundwater recharge disrupts the balance between virus and prokaryotic host communities, resulting in a deficiency of suitable prokaryotic host cells for viral proliferation.

## Introduction

Viruses infecting prokaryotes (members of the phylogenetic domains *Bacteria* and *Archaea*) are found in every aquatic habitat on Earth [1]. Viruses are obligate parasites and depend entirely on the metabolism of their hosts for proliferation. The first crucial step in the viral replication cycle is successful attachment and transfer of the viral genomic material into the host cell before the virus particle is degraded. Viruses have a certain preference for specific prokaryotic host taxa that they are able to recognize based on variations in, e.g., cell surface structures. Thus, any given virus can only infect a specific subset of prokaryotic taxa found in the same habitat; in the extreme, viruses may be species- or even strain-specific [2]. Viruses are incapable of directed movements and depend on stochastic processes (e.g., Brownian motion, host abundance; [3]) to meet their hosts for attachment and infection. In aquatic habitats, prokaryotes constitute the most abundant group of organisms [4]. Based on the nature of virus-host relationships detailed above one might argue that viruses proliferating in prokaryotic host cells generally outnumber other groups of viruses. Viral lysis converts particulate organic carbon into dissolved organic carbon and also liberates other nutrients with consequences for biogeochemical carbon cycling [5, 6]. Viruses have been shown to regulate prokaryotic diversity [7, 8] and have a profound effect on the genetic make-up of their prokaryotic host communities [1].

It has been estimated that around 60% of all microbial life on Earth is found in the continental subsurface including groundwater habitats [9, 10]. Yet the influence of viruses on subsurface biogeochemical cycling and microbial life is poorly understood. Previously, a number of authors have determined the abundance of viruses in a range of groundwater habitats including granite aquifers with viral abundance varying between 10^**5**^−10^**7**^ mL^**-1**^ [11], alluvial sand and gravel aquifers ranging from 10^**3**^−10^**8**^ viruses mL^**-1**^ [12–15, unpublished data by Pleyer et al. and Zhou et al. as reported in 16], springs with viral numbers from 10^**3**^−10^**6**^ mL^**-1**^ [17, 18], and other subsurface waters with 10^**4**^−10^**6**^ viruses mL^**-1**^ [19, 20]. However, to the best of our knowledge, direct measurements of virus production rates are completely missing for groundwater habitats. In the present study we used the virus-dilution approach [21] to measure lytic virus production in shallow groundwater at the bank of the Danube River at a site near Vienna (Austria) over a period of five months. The virus-dilution approach makes use of the fact that virus infection is density-dependent [3]. Thus, viruses are largely removed from the sample and the retained prokaryotic cells are incubated in virus-free sample water. Because the extreme dilution of viruses in the sample prevents new viral infections from occurring during the experimental incubations, any increase in viral abundance must be the result of viral infection processes already underway before the sample was taken. This approach was used successfully to quantify viral production from surface [22, 23] to deep-sea marine environments [24] and from estuarine [25] to anoxic waters [26]. In order to better understand the temporal dynamics of groundwater microbiology at the study site we also collected data on viral particle decay as well as on prokaryotic and viral abundance along with key physico-chemical parameters.

## Materials and methods

### Sampling and physico-chemical parameters

For this study, sampling permits were obtained from Verbund and from the department of Land and Forestry of the Klosterneuburg Abbey. In total, four water samples were taken from a groundwater well located in Kritzendorf, Austria (location of well: https://www.openstreetmap.org/?mlat=48.33997922578082&mlon=16.30808266553089#map=19/48.33998/16.30808) from a depth of about 10 m below land surface and one sample from the surface of the main stream of the nearby Danube River, approximately 45 m away from the well. Groundwater flow at the sampling site follows the elevation gradient from the hill to the valley and then follows the flow direction of the Danube River. The aquifer in the sampling area is characterized by alluvial sand and gravel deposits. During low and medium water levels oft the Danube River we expect a discharge of groundwater into the river. River water infiltrates quantitatively into the aquifer only at high water levels of the Danube. The water table depth was 3.87 m below land surface in July, 4.80 m below land surface in August, 3.98 m below land surface in October, and 4.45 m below land surface for the sampling in November 2020. The well was purged by pumping at least 160 L of water from the well (pump MP1, Grundfos), corresponding to twice the volume of the well itself, before 2 L of groundwater samples were transferred into a sampling bottle (polycarbonate, Nalgene). Sampling bottles were soaked in 0.5 N HCl overnight and rinsed twice with MilliQ water (Millipore) and actual sample before sample collection. Groundwater sampling was conducted on July 30^**th**^, August 25^**th**^, October 8^**th**^, and November 25^**th**^ 2020. During the sampling in November, we also sampled 2 L of surface water from the nearby Danube River. All samples were stored in plastic coolers to prevent temperature changes during the ~2 hours of transportation to the lab but without any special precautions to prevent changes in the concentration of dissolved oxygen. Additionally, pH, electrical conductivity (μSi cm^**-1**^), water temperature (°C), and the concentration of dissolved oxygen (mg L^**-1**^) were measured on site using field electrodes (WTW multiparameter sensor, Weilheim, Germany).

### Determining prokaryotic and viral abundance

Prokaryotic and viral abundance was determined by flow-cytometry as previously described [27, 28]. In particular, 1.8 mL of water sub-sampled in the lab from either the sampling bottles or the containers for the experimental incubations were fixed immediately with glutaraldehyde (0.5% final concentration; 111-30-8, Merck) at room temperature for 10 minutes, subsequently flash-frozen in liquid nitrogen, and maintained at -80°C until further analysis. Upon thawing, samples were diluted in a ratio of at least 1:2 in 1× Tris-EDTA buffer (1 M Tris, 0.5 M EDTA, pH 8.0) and the nucleic acids of prokaryotic cells and viruses were stained with SYBR Green I (S9430, Merck). Background fluorescence was corrected by using a 1:2 dilution of 1× Tris-EDTA buffer in MilliQ water as a blank instead of an actual sample. Finally, prokaryotic cells and viruses were enumerated flow-cytometrically (FACSAria III, BD Biosciences) on cytograms of side scatter versus green fluorescence.

### Filtration procedure

Immediately upon arrival in the lab, 1.8 mL subsamples were taken to enumerate prokaryotes and viruses as described above. Subsequently, water samples (1.5 L) were subjected to sequential tangential-flow filtration to obtain prokaryotic and virus concentrates as well as particle-free water from the same sample. In detail, water was first filtered using 0.22 μm pore-size tangential-flow filtration cartridges (Vivaflow 200, VF20P7, Sartorius) operated by a peristaltic pump (Console Drive, Master Flex) to concentrate prokaryotic cells (100−200 mL final volume, concentration factor 7.5−15 based on volumina), used subsequently as inoculum in the experimental incubations to determine virus production rates. The filtrate from this first filtration step, still containing viruses, was subjected to filtration using a tangential-flow filtration cartridge with a molecular weight cut-off of 100 kDa (Vivaflow, VF20P4, Sartorius). The virus concentrate from this second filtration step (100−200 mL final volume, concentration factor 7.5−15 based on volumina) was used to determine viral decay, whereas the filtrate, not containing any viruses or prokaryotic cells, was used as growth medium in subsequent experiments. Prior to filtrations, all tubings and cartridges were soaked in 70% ethanol overnight, rinsed with MilliQ water and 0.5 L of sample. In order to reduce excessive particle loads in the sample from the Danube River in November 2020, river water and, to ensure comparability, the corresponding groundwater sample were pre-filtered over membrane filters with a pore-size of 3 μm (Isopore, TSTP04700, Millipore) before sequential tangential-flow filtrations. Filtrations were performed on the lab bench, likely resulting in elevated concentrations of dissolved oxygen in groundwater samples as compared to in situ conditions.

### Determining virus production and viral decay

We used the virus-dilution approach [21] to determine virus production as previously described [26]. In detail, 5 mL of the prokaryotic concentrate obtained from groundwater samples were added to 45 mL of particle-free (100 kDa-filtered) water in 50 mL polypropylene tubes (Greiner). For the sample from the Danube River taken in November 2020, the volume of prokaryotic concentrate used in virus-dilution incubations was reduced to 2.5 mL in 47.5 mL particle-free filtrate due to higher abundances of prokaryotes and viruses in the stream versus groundwater. To determine viral decay in groundwater, parallel incubations containining 5 mL of virus concentrate in 45 mL of particle-free water were performed in July, August, and October. As for virus-dilution incubations, only 2.5 mL of virus concentrate were added to 47.5 mL of particle-free water for the sample from the Danube River obtained in November. Viral decay was not determined for the groundwater sample in November. In July, August, and October experimental incubations (virus-dilution approach and viral decay) were set-up in triplicates, whereas experiments from groundwater and river samples in November were performed in duplicates. All incubations were performed in the dark at in situ temperatures and sub-sampled every 4 hours to determine prokaryotic and viral abundance for a time span of 48 hours.

### Data treatment and statistical analyses

Data on the temporal development of prokaryotic and viral abundance of individual virus-dilution incubations were used to calculate virus production rates according to previously described equations [22]. Briefly, virus production was calculated as the slope between a local minimum (*V*_*min*_) and maximum (*V*_*max*_) in viral abundance. In case that more than one peak in viral abundance occurred during the incubation period, virus production was calculated based on the formula *virus production = [(V*_*max1*_ *–V*_*min1*_*) + (V*_*max2*_ *–V*_*min2*_*)]/(t*_*max2*_ *–t*_*min1*_*)*, where *t = time*. For the samples in July, August, and October, averages and standard deviations were calculated from triplicate incubations and for the sample in November the average values were calculated from duplicate incubations.

Viral abundance data obtained from all viral decay incubations corresponding to a single sample were fitted to the logistic equation *y = a × b*^*x*^, where *y* corresponds to viral abundance, *a* represents the *y*-axis offset, *b* is the decay coefficient and *x* represents time. We also calculated 95% confidence intervals for the estimated equation parameters to test if the calculated decay coefficients were significantly different from 1. Viral decay rates were not used to correct virus production rates.

Spearman rank correlation coefficients (*ρ*) were calculated to test for significant correlations between data on auxiliary parameters (prokaryotic and viral abundance, temperature, oxygen concentration, pH, and electrical conductivity) measured on site versus virus production or between prokaryotic and viral abundance. For correlations coefficients calculated between virus production and auxiliary parameters we have used a statistical re-sampling technique, i.e., virus production data from each individual incubation (triplicates for incubations in July, August, and October and duplicates in November) were compiled against the corresponding data on auxiliary parameters for each sampling campaign. This approach resulted in a sample size of 11, except for pH, where it was 8 due to the lack of data on pH in August. All data and statistical analyses were performed using the software Mathematica (version 13.2, Wolfram Research Inc.).

## Results

### Physico-chemical characteristics

From July−November 2020, temperature in groundwater at the sampling site varied from 13.2−14.9°C (average: 14.1°C) compared to 6.3°C in the Danube River adjacent to the groundwater well in November (S1A Fig). In groundwater, the variation of the concentration of dissolved oxygen was between 0.4−1.1 mg L^**-1**^ (average: 0.7 mg L^**-1**^) throughout the study period. In contrast, in the Danube River the concentration of oxygen was 12.1 mg L^**-1**^ in November (S1A Fig). The pH in groundwater varied between 7.13−7.52 (average: 7.30), whereas it was 8.10 in the Danube River in November (S1B Fig). Groundwater was characterized by an overall variation in conductivity between 495−590 μSi cm^**-1**^ (average: 543 μSi cm^**-1**^) during the study period. Based on conductivity it was possible to distinguish a summer situation from July−August with low electrical conductivity in groundwater varying between 495−513 μSi cm^**-1**^ from an autumn period from October−November with relatively high electrical conductivity from 573−590 μSi cm^**-1**^ (S1B Fig). Compared to groundwater, conductivity in the Danube River was much lower and determined to be 424 μSi cm^**-1**^ in November (S1B Fig). Overall, physico-chemical parameters in groundwater showed little variation throughout the study period with the exception of the concentration of oxygen (S2 Fig).

### Prokaryotes and viruses

#### Abundances

Prokaryotic abundance in groundwater varied from 9.4−16.7×10^**4**^ mL^**-1**^ during July−August and between 13.9−15.5×10^**4**^ mL^**-1**^ during October−November compared to 44.3×10^**4**^ mL^**-1**^ in the Danube River in November (S1C Fig). Variation of viral abundance during July−August was between 14.5−36.2×10^**4**^ mL^**-1**^ and during October−November from 19.7−22.0×10^**4**^ mL^**-1**^ (S1C Fig). In November, viral abundance was 596.2×10^**4**^ mL^**-1**^ in the Danube River; an order of magnitude higher compared to groundwater during the same month (S1C Fig). Based on these abundances, the virus-to-prokaryote ratio (VPR) in groundwater varied between 0.9−3.9 during July-August and was 1.4 in October and November as compared to 13.5 in the Danube River in November. In groundwater, prokaryotic and viral abundance were not correlated with each other (*ρ* = -0.8, *p* = 0.2, N = 4).

#### Virus production and viral decay

**Fig 1:**
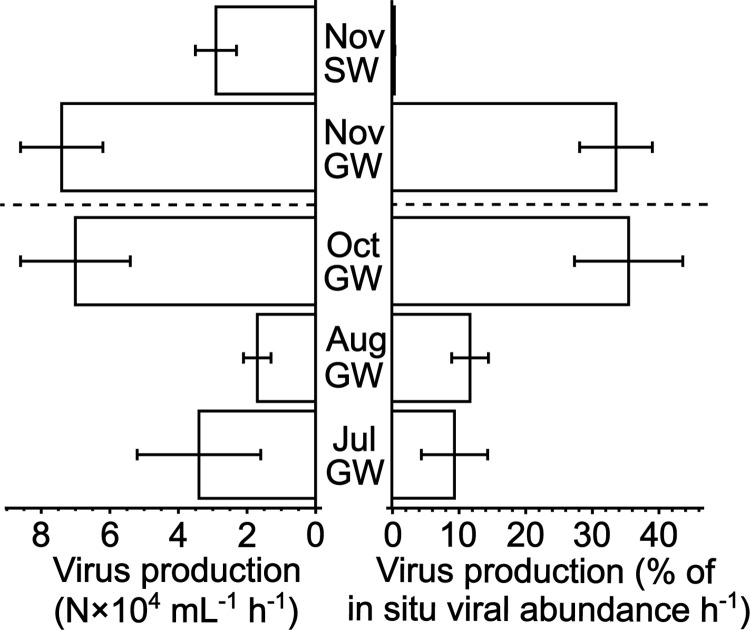
Virus production. The figure shows virus production (N×10^**4**^ mL^**-1**^ h^**-1**^) and virus production expressed as % of in situ viral abundance h^**-1**^ as determined in groundwater (GW) in July, August, October, and November as well as in surface water (SW) of the Danube River in November. Error bars represent standard deviations of triplicate incubations in July, August, and October and the range of duplicate incubations in November for groundwater (GW) and surface water of the Danube River (SW).

During the study period, virus production on average varied from 1.7−7.4×10^**4**^ mL^**-1**^ h^**-1**^ in groundwater and averaged 2.9×10^**4**^ mL^**-1**^ h^**-1**^ in the Danube River in November (Fig 1). In groundwater, virus production expressed as percent of in situ viral abundance h^**-1**^ averaged 9−12% of viral abundance h^**-1**^ in July−August and 34−36% of viral abundance h^**-1**^ in October−November (Fig 1). In contrast, in the Danube River virus production averaged only 0.5% of viral abundance h^**-1**^ in November (also see S1C Fig). Virus production in groundwater was significantly positively correlated with electrical conductivity but not with the concentration of dissolved oxygen (Table 1), despite its high coefficient of variation (S2 Fig). Viral decay could only be detected in groundwater in July and amounted to 3−5% of viral abundance h^**-1**^ (S3 Fig and S1 Table). Raw data for all experimental incubations are available online in the supporting information section (S1 Data).

**Table 1:** Spearman rank correlation coefficients (*ρ*) between virus production (VP) and physico-chemical parameters as measured in groundwater.

Parameter	Spearman’s *ρ*	*p*-value	*N*
**Prokaryotic abundance**	-0.10	0.35	11
**Viral abundance**	0.12	0.72	11
**Temperature**	-0.12	0.72	11
**Dissolved oxygen concentration**	-0.42	0.20	11
**pH**	-0.73	0.04	8
**Electrical conductivity**	**0.83**	**<0.01**	11

Spearman rank correlation coefficients were calculated between VP derived from individual incubations and corresponding prokaryotic and viral abundance, temperature, dissolved oxygen concentration, pH, and electrical conductivity measured in situ during groundwater sampling. Correlations with a significance level below 1% are marked in bold-face letters.

## Discussion

### Methodological considerations

DNA-containing membrane vesicles produced by prokaryotic cells similar in size to virus particles [29] have been implicated to alter viral abundance data determined flow-cytometrically from SYBR Green I-stained samples as used in this study [28]. The first counting technique developed for viruses in aquatic habitats uses transmission electron microscopy and relies on visually-identifying viruses in the sample [30]; thus, this technique does not suffer from biases due to the presence of DNA-containing membrane vesicles as these are not identified as being of viral origin. Later techniques such as epifluorescence microscopy and flow-cytometry were initially cross-calibrated with each other and consistently yield data that correspond well with each other [28, 31]. The finding that all three direct-counting techniques yield similar results with the earliest technique not being susceptible to confuse prokaryotic membrane vesicles with virus particles is a strong argument against biased viral abundance data obtained by flow-cytometry. Additionally, the refractive index difference between prokaryotic membrane vesicles and the sheath fluid used in flow-cytometry is very small making it very difficult to even detect membrane vesicles on commercially-available flow-cytometers [32]. Furthermore, only a small fraction of prokaryotic membrane vesicles contain dsDNA and those that do, often have very little [33]. In summary, we are confident that our virus abundance data are not meaningfully contaminated by the potential presence of dsDNA-containing prokaryotic membrane vesicles.

The ability to detect viruses in aquatic samples by flow-cytometry does not depend so much on the size of the viral particle, instead fluorescence intensity after staining the viral genome with SYBR Green I determines if a given virus particle can be detected. For viruses infecting prokaryotes, the viral genomic material is literally squeezed into a small capsid and SYBR Green I’s ability to bind to the viral genome depends on how dense the genome is packaged. There are taxon-specific differences in how dense virus genomes are packaged inside prokaryotic virus capsids [34] resulting in the finding that very small viruses can have a high fluorescence intensity compared to physically much larger viruses with a low fluorescence signal (e.g., see Fig 1 in [35]). For that reason, fluorescence intensity of stained viruses cannot be used to draw conclusions about the size of the virus particle or its genome. SYBR Green I preferentially binds to double-stranded DNA. Given that eukaryotic single-stranded DNA viruses might be an important component of groundwater virus communities [17] it is important to mention that these viruses will largely be ignored by flow-cytometry as performed here.

### Viruses in a physically-constrained environment

#### Unconnected prokaryotic and viral abundances

With ~10^**5**^ viruses mL^**-1**^ (S1C Fig) and virus-to-prokaryote ratios averaging 1.9 in groundwater throughout the study period, our data are well within the range of previously reported data for alluvial sand and gravel aquifers of 10^**3**^−10^**8**^ viruses mL^**-1**^ [12–15, unpublished data by Pleyer et al. and Zhou et al. as reported in 16]. In most aquatic surface habitats, prokaryotic and viral abundance show a positive correlation with each other over spatial and temporal gradients [36, 37]. Viruses are obligate parasites, generally viral infection is density-dependent [3], and viral abundance is the result of the balance between virus production and viral particle decay. Thus, positively-correlated prokaryotic and viral abundances can be considered a hallmark sign for a lytically-reproducing viral community mostly targeting prokaryotic host cells. In fact, viral and prokaryotic abundances were found to correlate positively with each other in some groundwater habitats [11, 13] while in others no correlation between the numbers of viruses and prokaryotes were found (unpublished data by Pleyer et al. as reported in [16]). In the present study, prokaryotic and viral abundance was not correlated with each other in groundwater during the study period (see **Results** section). This suggests a priori that most viruses in groundwater may have had an origin that is not compatible with in situ lytic virus production by free-living prokaryotic host cells. Yet, in the following we point-out several substantial challenges for viral proliferation in the groundwater environment that demand attention before interpreting the data at hand.

#### Intrinsic viral decay

The decay of viral particles measured in this study reflects intrinsic decay, specifically, the decay attributed to the inherent properties of the virus particles themselves. It should be mentioned that other factors contributing to virus particle decay have been excluded through incubation in the dark and pre-experimental filtration procedures [38−41]. The intrinsic decay of viruses infecting prokaryotes is influenced by the pressure build-up within their capsids. This pressure is due to the genomic material packaged inside the virus capsid, which must be counterbalanced by the capsid to maintain the integrity of the virus particle. Different virus taxa vary widely in terms of capsid thickness, capsid size, and size of the genome [34]. This results in considerable variation in intrinsic viral decay among different virus taxa, i.e., the duration between the release of viral progeny from a lysed host cell until the structural failure of these viruses due to internal pressure [34]. Thus, at least over a two-day period and in the absence of external viral decay-facilitating factors, viruses found in groundwater at our study site (and in the Danube River in November) appeared to be rather stable (S3 Fig and S1 Table). These findings are in line with previous studies showing that virus particles in groundwater appear to be stable for months to years [42].

#### Obstacles to virus-host encounter

Viral abundance in groundwater throughout the sampling period was low (S1C Fig) and appeared to be in contrast to the high virus production rates of 9−12% of viral abundance h^**-1**^ in summer and of 34−36% of viral abundance h^**-1**^ in autumn (Fig 1). This discrepancy cannot be explained by viral particle decay, which, if detectable at all, was small compared to virus production rates (S1C Fig and S1 Table). A decisive step in the reproductive cycle of any virus is meeting its proper host before the virus particle disintegrates or is otherwise rendered non-infective [38−41]. Viruses are incapable of directed movements, thus, physical contact between a virus and its proper host is diffusion-limited and among other parameters depends on the abundance of both the virus particles and the potential host cells [3]. The most obvious difference between surface and groundwater environments is the fact that groundwater moves within a matrix of porous sediment material, the aquifer; at the study site the groundwater aquifer is composed of alluvial sand and gravel sediments. Sediment layers are typically more compact than the water column, which limits the movement of particles and dissolved molecules. Lower diffusion rates in sediments are due to interstitial spaces between sediment particles and potential interactions with solid surfaces in the sediment layers. In comparison, the water column provides a more open environment for particles and molecules to move freely, resulting in higher diffusion rates. This has fundamental consequences for virus-host encounter rates: the lower diffusion rates in groundwater together with the potential of viruses adsorbing to the huge surface area of the aquifer materials before a proper host can be met greatly reduces virus-host-encounter rates compared to the open water column. In fact, adsorption of viruses onto the surface of particles is a well described reduction mechanism for viruses, whereby virus particles are effectively removed from the potentially infective virus community [39, 40]. This also means that similar virus production rates measured at the surface and in groundwater need to be interpreted fundamentally different as a much smaller fraction of progeny viruses may actually infect new host cells in groundwater compared to surface conditions. It needs to be mentioned that changes in the concentration of dissolved oxygen due to the filtration procedure might have influenced virus production in groundwater. Under the assumption that the effect of changes in the concentration of dissolved oxygen in samples with initially low levels of oxygen will be stronger compared to samples with higher concentrations of dissolved oxygen, we would expect a relationship with the concentration of dissolved oxygen and virus production. However, we did not find any evidence that this was the case (Table 1, Fig 1 and S1A Fig) despite substantial variation of the concentration of dissolved oxygen in groundwater (S2 Fig). A scenario where a substantial fraction of progeny viruses is scavenged by the aquifer sedimentary matrix at the study site may explain the discrepancy between substantial virus production rates that are not reflected by in situ viral abundances (Fig 1, S1A Fig and Table 1) versus hardly any detectable intrinsic viral decay (S3 Fig and S1 Table). Such a scenario might also explain the substantially lower virus-to-prokaryote ratios found in groundwater as opposed to the Danube River (see **Results** section).

### Seasonality in groundwater virus production

Environmental variability in groundwater cannot be neglected and appears to also have been influencing prokaryotic and viral abundance as well as virus production at the study site (S1C and S2 Figs and Fig 1). Most notably, we found a strong positive and statistically significant correlation between electrical conductivity and virus production (Table 1). Electrical conductivity may be used as an indicator for the level of dilution of groundwater. Thus, comparatively high levels of electrical conductivity are found in groundwater that had a longer time to get enriched with ions dissolved from the geological background increasing electrical conductivity, whereas lower levels of electrical conductivity indicate the dilution of groundwater by external sources such as seeping precipitation or, in the case of our study site, water from the Danube River. Groundwater recharge likely was driven by a combination of precipitation and the water level of the nearby Danube River, however, publicly available data are inconclusive (https://www.noe.gv.at/wasserstand/#/de/Messstellen; S4 Fig). Regardless of the exact mechanism of groundwater recharge, based on conductivity it is possible to identify a summer situation from July−August with low electrical conductivity from an autumn period during October−November with relatively high electrical conductivity (see **results** section; S1B Fig). A similar temporal dichotomy was also clearly identifiable in virus production expressed as % of in situ viral abundance h^**-1**^ (Fig 1). In other words, higher groundwater recharge during summer of 2020 coincided with low virus production as compared to autumn with little groundwater recharge and ~3.5-times higher virus production rates. Although virus infection is density dependent [3], the observed seasonality in virus production (Fig 1) cannot be explained by changes in abundances of prokaryotes and/or viruses (Table 1).

Viruses infecting prokaryotes do have a certain degree of specificity for their host cells [2]. Thus, enhanced groundwater recharge in summer might have altered the community composition of prokaryotes indirectly by changing the availability of nutrients causing changed growth patterns of specific prokaryotic taxa, in turn lowering virus production due to an imbalance between virus and host communities. Alternatively, groundwater recharge may have changed prokaryotic and viral communities directly by importing taxa not normally found in groundwater or that were ill adapted to the environmental conditions underground similarly resulting in low virus production rates due to a shortage of suitable hosts. By autumn, the effects of the higher groundwater recharge during summer may have subsided, allowing prokaryotic and virus communities to mature and adapt to each other and the environmental conditions, resulting in ~3.5-times higher virus production rates as compared to summer (Fig 1).

## Conclusions

Our data clearly show that lytic virus production from free-living prokaryotic hosts actually occurs in groundwater at the study site. The lack of a correlation between prokaryotic and viral abundance in groundwater throughout the study period is best explained by substantial virus particle adsorption to the huge surface area of the aquifer materials. These virus adsorption losses appear to be compensated by considerably higher levels of virus production rates than would otherwise be necessary to maintain viral numbers at the study site. Seasonality due to different levels of groundwater recharge as detailed by differences in electrical conductivity have an immediate influence on levels of virus production rates. At the study site, low levels of groundwater recharge in autumn 2020 coincided with ~3.5-times higher virus production rates as compared to summer with higher groundwater recharge. The data are best explained by a scenario where groundwater recharge alters the balance between virus and prokaryotic host communities in a way that leads to a shortage of suitable host cells for viral proliferation.

## Supporting information

S1 Fig Temperature, concentration of dissolved oxygen, pH, electrical conductivity, prokaryotic and viral abundance. The figure shows (A) temperature (°C) and the concentration of dissolved oxygen (mg L^**-1**^), (B) pH and electrical conductivity (μS cm^**-1**^), and (C) prokaryotic and viral abundance (both in N×10^**4**^ mL^**-1**^) as measured in groundwater (GW) in July, August, October, and November as well as in surface water (SW) of the Danube River in November. Note that the x-axis for viral abundance (C) is divided into two scales due to the large differences between groundwater and surface water viral abundances.
(PDF)

S2 Fig Coefficient of variation. The figure show the coefficient of variation for temperature, the concentration of oxygen, pH, electrical conductivity, prokaryotic and viral abundance, and virus production as measured in groundwater at the study site. The error bar for the coefficient of variation of virus production represents the standard deviation and indicates that virus production was measured in triplicates (July, August, October) and duplicates (November) as compared to the other parameters that were measured once per sampling.
(PDF)

S3 Fig Viral decay. Temporal development of viral abundance within incubations to determine viral particle decay in groundwater in (A) July, (B) August, (C) October, and (D) in surface water of the Danube River in November. Error bars show the standard deviation of triplicate incubations in groundwater in July, August, and October (A−D); error bars for data from surface water in November (D) depict the range of duplicate incubations. Data were fitted to the logistic function *y = a × b*^*x*^, where *y* stands for viral abundance, *a* represents the *y*-axis offset, *b* is the decay coefficient and *x* represents time. Additionally, dashed lines represent 95% confidence limits for the fitted data as detailed in (S1 Table).
(PDF)

S4 Fig Precipitation and water level of the Danube River. (A) Daily precipitation levels recorded at the station "Hohe Warte" and (B) the water level of the Danube River at the station "Korneuburg" during the sampling period in 2020. As comparison, electrical conductivity of groundwater as measured at the sampling site is displayed in both panels to judge the influence of either precipitation or water level of the Danube River on groundwater recharge.
(PDF)

S1 Data Prokaryotic and viral abundance from incubations. This spreadsheet file provides original data on the temporal development of prokaryotic and viral abundance from the experimental incubations sorted for each sampling date.
(XLS)

S1 Table Viral decay based on viral abundance data fitted to the logistic decay function *y = a×b*^*x*^. (PDF)
