# Supplementary figures and images for: Virus production in shallow groundwater at the bank of the Danube River
Source: PLoS One. 2024 Aug 29;19(8):e0306346. doi: 10.1371/journal.pone.0306346 (PMC11361564; doi:10.1371/journal.pone.0306346)

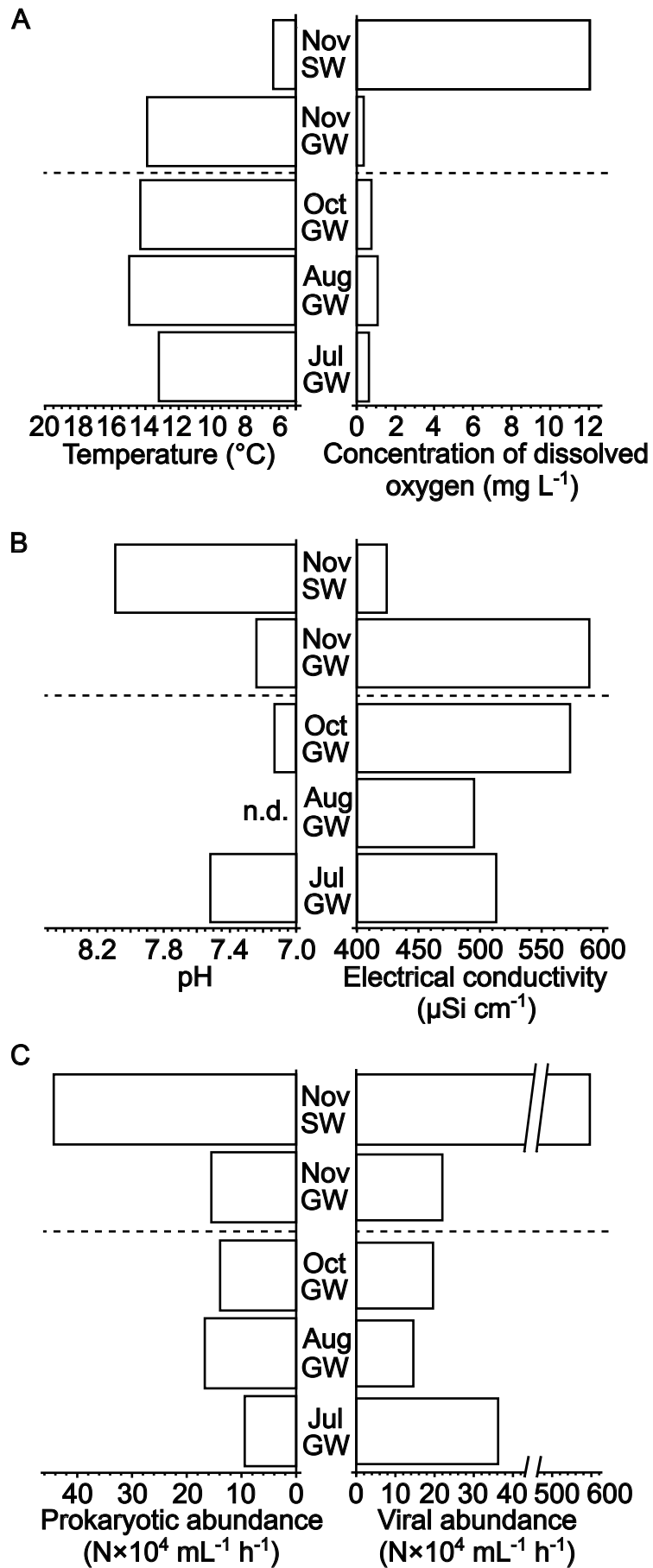

S1 Figure

Supplement: S1 Fig — The figure shows (A) temperature (°C) and the concentration of dissolved oxygen (mg L-1), (B) pH and electrical conductivity (μS cm-1), and (C) prokaryotic and viral abundance (both in N×104 mL-1) as measured in groundwater (GW) in July, August, October, and November as well as in surface water (SW) of the Danube River in November. Note that the x-axis for viral abundance (C) is divided into two scales due to the large differences between groundwater and surface water viral abundances. (PDF) [file pone.0306346.s001.pdf]

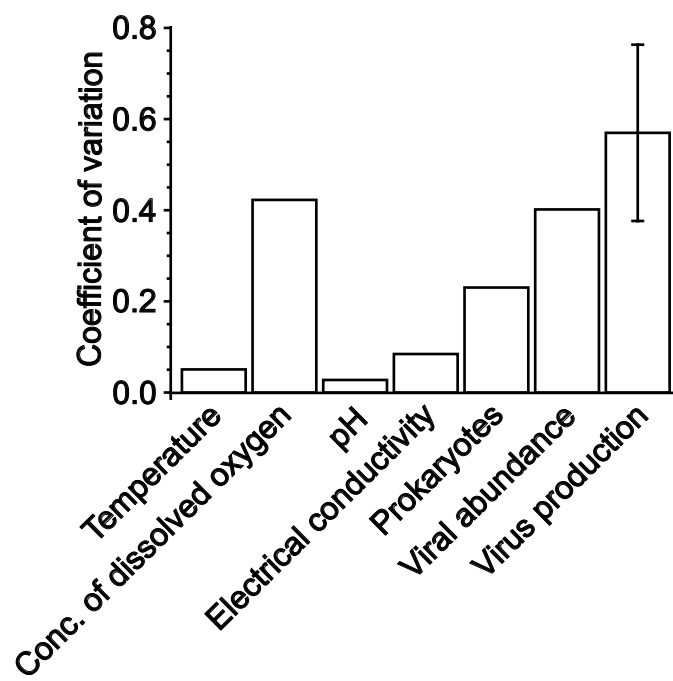

S2 Figure

Supplement: S2 Fig — The figure show the coefficient of variation for temperature, the concentration of oxygen, pH, electrical conductivity, prokaryotic and viral abundance, and virus production as measured in groundwater at the study site. The error bar for the coefficient of variation of virus production represents the standard deviation and indicates that virus production was measured in triplicates (July, August, October) and duplicates (November) as compared to the other parameters that were measured once per sampling. (PDF) [file pone.0306346.s002.pdf]

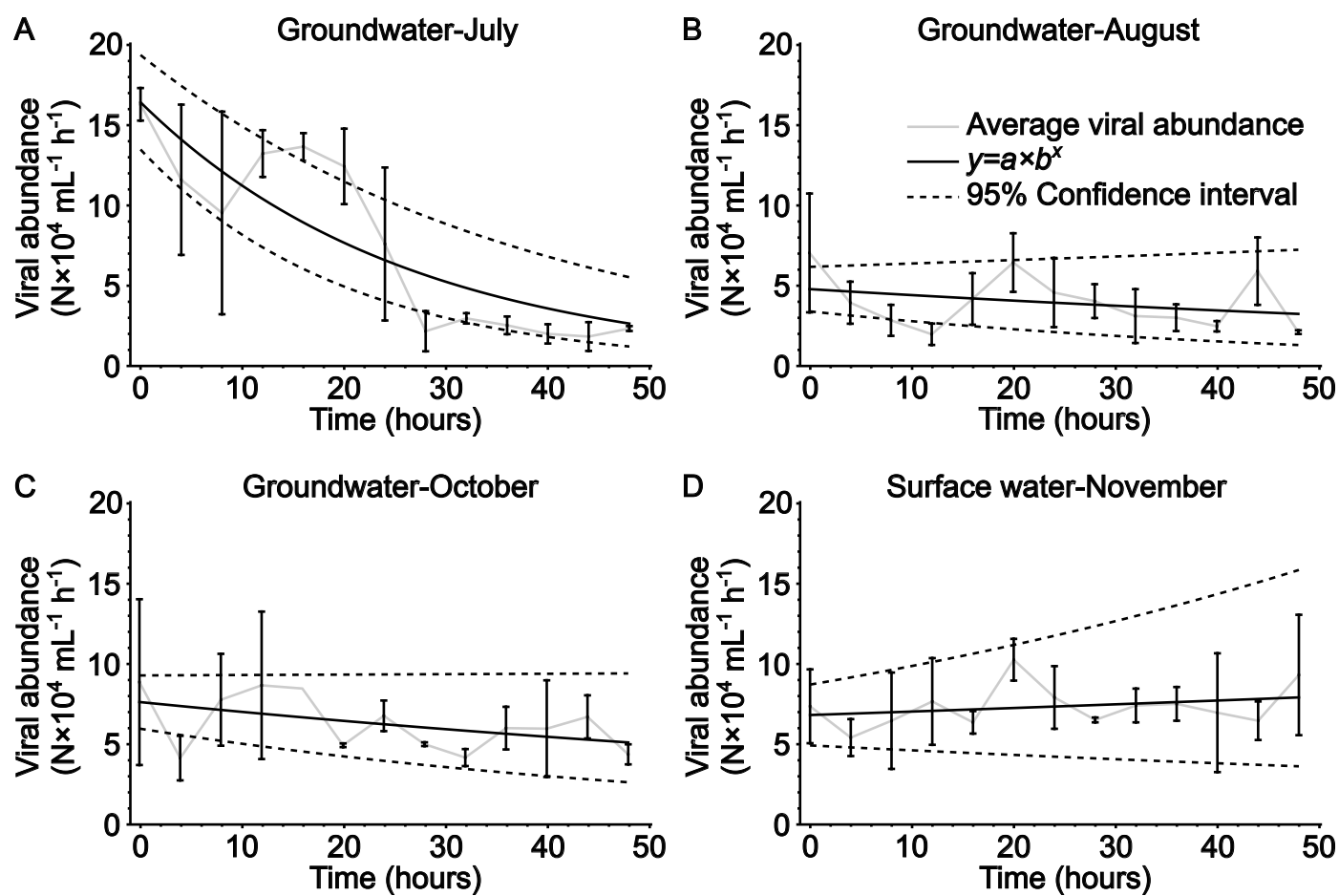

S3 Figure

Supplement: S3 Fig — Temporal development of viral abundance within incubations to determine viral particle decay in groundwater in (A) July, (B) August, (C) October, and (D) in surface water of the Danube River in November. Error bars show the standard deviation of triplicate incubations in groundwater in July, August, and October (A−D); error bars for data from surface water in November (D) depict the range of duplicate incubations. Data were fitted to the logistic function y = a × bx, where y stands for viral abundance, a represents the y-axis offset, b is the decay coefficient and x represents time. Additionally, dashed lines represent 95% confidence limits for the fitted data as detailed in (S1 Table). (PDF) [file pone.0306346.s003.pdf]

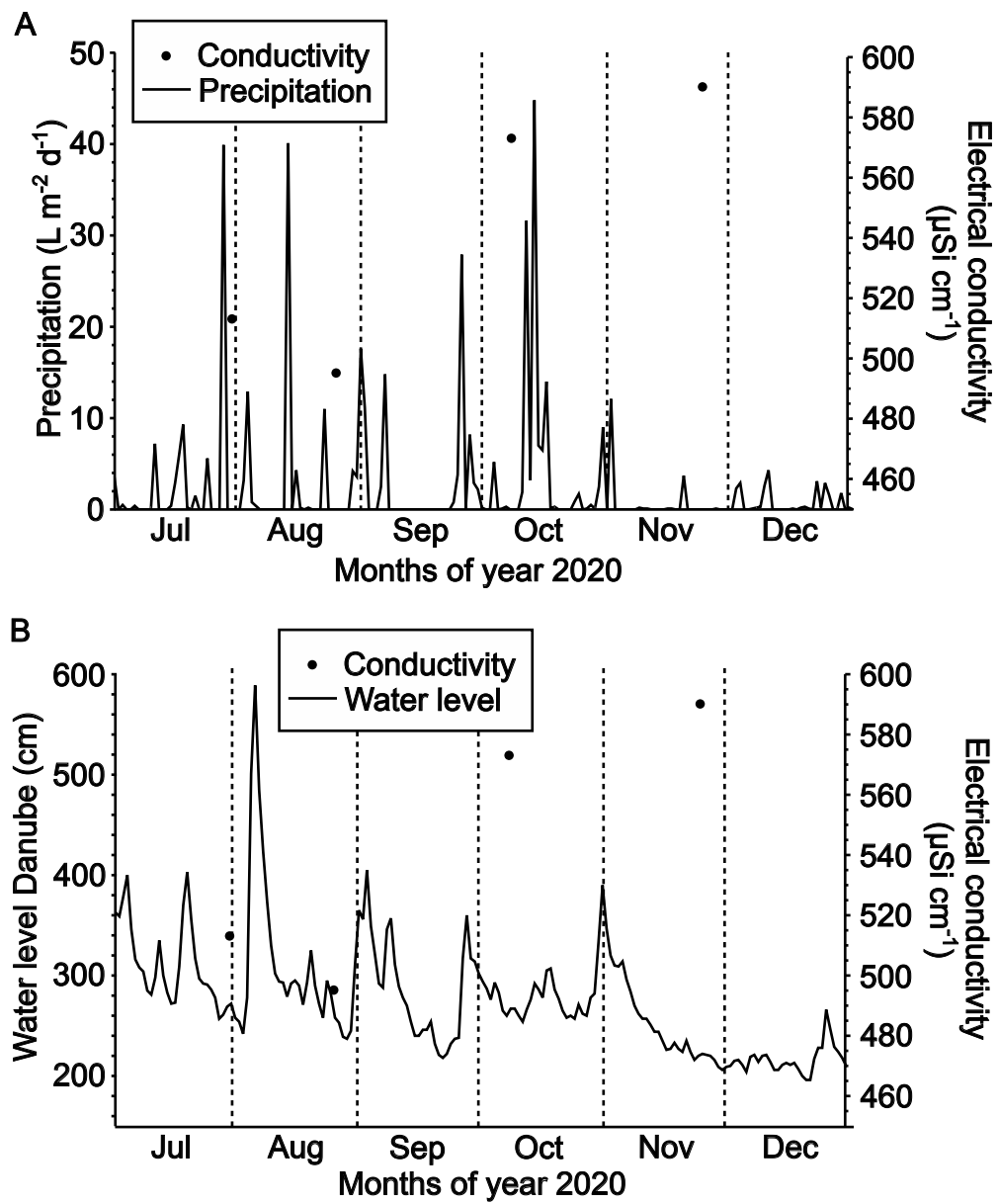

S4 Figure

Supplement: S4 Fig — (A) Daily precipitation levels recorded at the station "Hohe Warte" and (B) the water level of the Danube River at the station "Korneuburg" during the sampling period in 2020. As comparison, electrical conductivity of groundwater as measured at the sampling site is displayed in both panels to judge the influence of either precipitation or water level of the Danube River on groundwater recharge. (PDF) [file pone.0306346.s004.pdf]
