# Supplementary material for: Virus production in shallow groundwater at the bank of the Danube River
Source: PLoS One. 2024 Aug 29;19(8):e0306346. doi: 10.1371/journal.pone.0306346 (PMC11361564; doi:10.1371/journal.pone.0306346)
Supplement: S1 Table — (PDF) [file pone.0306346.s006.pdf]

S1 Table. Viral decay based on viral abundance data fitted to the logistic decay function $y=a \times b^x$.

Sampling	*a*	*p*-value	Interval	*b*	*p*-value	Interval
July	16.43	<0.0001	13.49−19.37	0.96	<0.0001	0.95−0.97
August	4.85	<0.0001	3.46−6.23	0.99	<0.0001	0.98−1.00
October	7.66	<0.0001	6.00−9.32	0.99	<0.0001	0.98−1.00
Danube-November	6.86	<0.0001	4.96−8.75	1.00	<0.0001	0.99−1.01

The table gives the parameters *a* (*y*-axis intercept) and *b* (rate of decay) as estimated from viral abundance data (*y*) of experiments used to measure viral decay over time (*x*). Additionally, 95% confidence intervals are presented as well. See also S5 Figure.
